# Sex differences in Tfh cell help to B cells contribute to sexual dimorphism in severity of rat collagen-induced arthritis

**DOI:** 10.1038/s41598-020-58127-y

**Published:** 2020-01-27

**Authors:** Mirjana Dimitrijević, Nevena Arsenović-Ranin, Duško Kosec, Biljana Bufan, Mirjana Nacka-Aleksić, Ivan Pilipović, Gordana Leposavić

**Affiliations:** 1 Department of Immunology, Institute for Biological Research “Siniša Stanković”, University of Belgrade, Bulevar despota Stefana 142 Belgrade, Serbia; 2 Department of Microbiology and Immunology, Faculty of Pharmacy, University of Belgrade, Vojvode Stepe 450 Belgrade, Serbia; 3 Immunology Research Center “Branislav Janković”, Institute of Virology, Vaccines and Sera “Torlak”, Vojvode Stepe 458 Belgrade, Serbia; 4 Department of Pathobiology, Faculty of Pharmacy, University of Belgrade, Vojvode Stepe 450 Belgrade, Serbia

**Keywords:** Autoimmunity, Rheumatic diseases

## Abstract

The study examined germinal centre (GC) reaction in lymph nodes draining inflamed joints and adjacent tissues (dLNs) in male and female Dark Agouti rat collagen type II (CII)-induced arthritis (CIA) model of rheumatoid arthritis. Female rats exhibiting the greater susceptibility to CIA mounted stronger serum CII-specific IgG response than their male counterparts. This correlated with the higher frequency of GC B cells in female compared with male dLNs. Consistently, the frequency of activated/proliferating Ki-67+ cells among dLN B cells was higher in females than in males. This correlated with the shift in dLN T follicular regulatory (Tfr)/T follicular helper (Tfh) cell ratio towards Tfh cells in females, and greater densities of CD40L and CD40 on their dLN T and B cells, respectively. The higher Tfh cell frequency in females was consistent with the greater dLN expression of mRNA for IL-21/27, the key cytokines involved in Tfh cell generation and their help to B cells. Additionally, in CII-stimulated female rat dLN cell cultures IFN-γ/IL-4 production ratio was shifted towards IFN-γ. Consistently, the serum IgG2a(b)/IgG1 CII-specific antibody ratio was shifted towards an IgG2a(b) response in females. Thus, targeting T-/B-cell interactions should be considered in putative further sex-based translational pharmacology research.

## Introduction

Rheumatoid arthritis (RA) is a chronic autoimmune systemic inflammatory disease characterized by synovial inflammation and the progressive destruction of joint cartilage and bones significantly reducing health-related quality of life^1^. The prevalence of RA is approximately threefold higher in women than in men, and the disease in women exhibits a more severe course^2^. Thus, research focused on sex as a determinant of disease severity could be important for further personalized management of patients with this autoimmune disease^3^.

Collagen-induced arthritis (CIA) is a well-established experimental model mimicking many aspects of RA immunopathogenesis^4^. Differently from most of the mouse models of experimentally induced arthritis^5,6^, in several studies, female rats exhibited greater susceptibility to CIA compared with their male counterparts^7–10^. Thus, these rat CIA models seem to be suitable for research aimed to elucidate cellular and molecular mechanisms underlying sex differences in pathogenesis and clinical outcome of RA, as a base for designing efficient sex-specific therapies.

Sex differences in susceptibility to autoimmune diseases have been related to sex-based differences in immunopathogenesis and susceptibility of the target tissue to damage caused by autoimmune or inflammatory burden^11,12^. These differences most likely arise from intricate interactions between circulating sex steroids, microbiota, genetic and environmental factors^11,13^.

CIA is shown to be T helper (Th)1/Th17 mediated disease^14^. The main function of Th cells is to activate macrophages and fibroblasts and transform them into tissue-destructive cells^15^. Our previous studies in Dark Agouti (DA) rat CIA model showed more robust Th1/17 responses in female rats (exhibiting the higher incidence and the more severe CIA) compared with their male counterparts and pointed out to some underlying mechanisms^9,10^. However, although CD4+ T cells are of essential significance for the induction and maintenance of inflammation in CIA and RA, the activation of self-antigen-specific B cells is thought to be extremely important for the target tissue damage^16^. The role of B cells in the pathogenesis of CIA, as in RA, is likely to be complex^17^.

B cells contribute to RA development by producing antibodies specific for self-proteins, and/or altered self-proteins, such as collagen type II (CII), Fc portion of IgG (rheumatoid factor) and citrullinated protein antigens^18^. To corroborate the importance of antibodies in the pathogenesis of CIA are data indicating that the absence of B cells in genetically modified mice^19^ and lowered number of B cells in mice treated with anti-CD20 antibodies^20^, inducing lack of CII-specific autoantibodies, protected mice against CIA^19^. The pathogenic role of B cells in CIA is mainly attributed to the secretion of anti-collagen antibodies^17^. The CII-specific autoantibodies are crucial for both the initiation and perpetuation of the clinical disease^21^. These antibodies activate complement and/or residential and infiltrated cells carrying Fcγ receptor (FcγR) in the synovia to produce inflammatory cytokines, mediators and enzymes damaging cartilage and bone^21^. The severity of tissue damage and inflammation is largely determined by the outcome of interactions between the autoantibodies and complement and/or FcγR bearing cells^21^. This interaction depends on the antibody class/subclass profile (as different IgG subclasses differ in their capacity to bind FcγR or to activate complement) and their titres, as well as on the abundance of FcγR bearing inflammatory cells at the site of immune complex deposition^21^. It is also noteworthy that in the rat IgG class switching mainly depends on IFN-γ/IL-4 production ratio^22,23^. However, IL-17 can also participate in this regulation, as IL-17-deficient mice upon immunization with collagen exhibited the impaired capacity of collagen-specific IgG2a antibody generation^24^.

Germinal centres (GCs) provide a proper microenvironment for activation, somatic diversification, and affinity maturation of (auto)reactive B lymphocytes which occurred before the production of (auto)antibodies^25^. Mice that were selectively deficient in GCs are almost fully protected against CIA, whereas they are fully susceptible to collagen antibody-induced arthritis^17^. Following the induction of CIA GCs develop in inflamed joint tissues and secondary lymphoid tissues, including the lymph nodes draining inflamed joints and adjacent tissues (dLNs), often referred to as reactive lymph nodes^26^. An accumulating body of evidence suggests an important role of dLNs in the development and progression of CIA and RA trough cross-talk with the inflamed tissues, so peripheral tissues and their dLNs operate as a functional pathogenic unit^26^. Of note, Th cells from GCs referred to as follicular helper T (Tfh) cells critically contribute to the proliferation of (auto)antigen-primed GC B cells, isotype switching, and their somatic hypermutations leading to the generation of long-lived plasma cells and memory B cells^27^. Conventional CD4+ T cells differentiate into Tfh cells through a specific pathway that terminates in the high expression of the B-cell follicle homing chemokine (C-X-C motif) receptor 5 (CXCR5) and their localization to the GCs^27,28^. Thus, the stringent control of Tfh cell generation and function is critically important for the induction of an optimal humoral response against thymus-dependent antigens and the prevention of self-reactivity, alike^29,30^. Consistently, deregulations of Tfh response can contribute to the production of pathogenic autoantibodies, and thereby the promotion of autoantibody-mediated autoimmune diseases, including RA^29,30^. To corroborate this notion, in CIA, T-cell-specific CXCR5 deficiency results in a significant reduction in the formation of GCs leading to the decreased levels of collagen-specific antibodies and the resistance to CIA induction^25^. On the other hand, a subset of CD4+ Foxp3+ regulatory T cells expressing Tfh cell-associated molecules, including CXCR5, termed T follicular regulatory (Tfr) cells plays a critical role in the controlling Tfh cell activity, and thereby indirectly GC B-cell activity and the magnitude of (auto)antibody production^27^. However, Tfr cells may exhibit a direct regulatory action on GC B cells^31,32^. Thus, as Tfh and Tfr cells act as reciprocal and antagonistic regulators of B-cell responses, their balanced action is suggested to be essential for immune homeostasis^29^. Consistently, aberrant or disordered Tfh/Tfr cell balance may cause the loss of immune tolerance and consequently enhanced production of autoantibodies^29,33,34^.

To the best of our knowledge, data on sex differences in the antibody response in either RA or CIA are extremely limited. However, it has been shown that sex differences in B-cell signature in healthy subjects underlie disparities in incidence and course of some other antibody-mediated autoimmune diseases^35^. Most of immune related genes differentially expressed between sexes are significantly upregulated in B cells from females compared to males^35^. Generally, females exhibit higher basal serum immunoglobulin levels and mount greater antibody responses to antigens than males^36,37^.

Considering all the aforementioned, the study was designed to examine: i) putative sex differences in the magnitude of the total CII-specific IgG response in sera from DA rats affected by CIA, and its subclass profile, and ii) contribution of Th cell-dependent dLN GC reaction to IgG response in this model of RA.

## Materials and Methods

### Animals

The study included 22 male (250–280 g) and 22 female (180–210 g) four-month-old DA rats from the Immunology Research Center “Branislav Janković” animal facility (Belgrade, Serbia), authorized by The Ministry of Agriculture, Forestry and Water Economy of the Republic of Serbia. Animals (8 immunized rats/sex, in two independent experiments, and 6 non-immunized rats/sex included in one of these two experiments) were kept in polyethylene cages in an environment under standardized conditions regarding light, temperature, and humidity. Rats were fed on a standard food diet for rats and had access to tap water *ad libitum*. Animal care staff and veterinarian inspected the healthiness of rats on a daily basis. Design and methods applied in animal research were performed in keeping with the Directive 2010/63/EU of the European Parliament and of the Council on the protection of animals used for scientific purposes and the governmental regulations (The Law on Animal Welfare, “Official Gazette of Republic of Serbia”, no. 41/2009) and approved by The Ministry of Agriculture, Forestry and Water Economy of the Republic of Serbia (permit no. 323-07-01577/2016-05/14). The ARRIVE guidelines for reporting animal research were fully implemented in the study.

### Induction and clinical assessment of CIA

CIA was induced in rats anesthetized with a ketamine/xylazine cocktail by intradermal injection of 300 μg of bovine CII (Sigma-Aldrich Chemie GmbH, Taufkirchen, Germany) in incomplete Freund’s adjuvant (IFA) at the rat tail base as described^10^. From the 7^th^ day post-immunization (d.p.i.) until the 21^st^ d.p.i. rats were inspected for the development of arthritis daily. In brief, each paw was scored individually by the scoring system in which 1 point was given for inflamed metacarpophalangeal/metatarsophalangeal or interphalangeal joints of each toe, whereas 5 points were given to an inflamed wrist/ankle^38^. The maximum score for each paw was 15, providing the highest arthritis score of 60. The researchers (MD, NAR) assigned to evaluate the disease development were blinded to the experimental design.

### Preparation of cells

On the 21^st^ d.p.i. dLNs (popliteal) were isolated from rats deeply anesthetized with ketamine/xylazine cocktail, as previously described in detail^10^. For immunolabeling and cell cultures, single cell suspensions were prepared by passing dLN tissue through 70 μm nylon cell strainer (BD Biosciences, Erembodegem, Belgium) in Petri dishes with ice-cold FACS buffer [phosphate buffered saline (PBS) supplemented with 2% fetal calf serum (FCS, Gibco, Grand Island, NY, USA) and 0.01% NaN_3_ (Sigma-Aldrich Chemie GmbH)] or culture medium [RPMI 1640 medium (Sigma-Aldrich Chemie GmbH) supplemented with 2 mM l-glutamine (Serva, Heidelberg, Germany), 1 mM sodium pyruvate (Serva), 100 units/ml penicillin (ICN, Costa Mesa, CA, USA), 100 μg/ml streptomycin (ICN) and 10% FCS], respectively. Viable cells were counted in each suspension using improved Neubauer hemacytometer and trypan blue dye for the exclusion of non-viable cells.

### Purification of dLN B cells

For measurement of T-bet mRNA levels, B cells (CD45RA+ cells) were separated from dLN mononuclear cell suspensions using magnetic-activated cell sorting (MACS). Briefly, dLN cells resuspended in degassed PBS containing 0.5% bovine serum albumin (BSA) and 2 mM ethylenediaminetetraacetic acid (MACS buffer) were incubated with rat CD45RA microbeads (clone OX-33; Miltenyi Biotec, Gladbach, Germany) for 15 min at 4 °C. Next, the cells were washed in MACS buffer and magnetically-labeled cell fraction was positively selected using LS type columns and QuadroMACS separator, as instructed by the manufacturer (Miltenyi Biotec). Purity check showed CD45RA+ cell enrichment of >90% in resulting suspensions.

### Immunolabeling and flow cytometry analysis (FCA)

Immunostaining of lymph node cells and FCA were performed as previously described^10^. In brief, for surface immunolabeling, dLN cells were incubated with either fluorochrome-conjugated or unconjugated/biotin-conjugated monoclonal antibodies, followed by incubation with a second step reagent.

For intracellular cytokine staining, dLN cells were cultivated in culture medium with phorbol 12-myristate 13-acetate, ionomycin and brefeldin A (4 h at 37 °C in 95% air–5% CO_2_), harvested and subjected to immunolabeling. Intracellular staining for cytokine expression in *in vitro* restimulated cells and Foxp3 and Ki-67 expression in freshly isolated dLN cells was performed following surface immunostaining and overnight fixation/permeabilization with reagents from eBioscience in compliance with manufacturer’s instructions. Between the steps, cells were washed with permeabilization buffer (eBioscience). A list of monoclonal antibodies and second step reagents used in FCA is given in Supplementary Table S1.

Data were acquired on FACSCalibur flow cytometer (Becton Dickinson, Mountain View, CA, USA) and analysed by an examiner blinded for animal sex using FlowJo software version 7.8. (TreeStar Inc, Ashland, OR, USA) for the frequency of marker positive cells, and the changes in mean fluorescence intensity (MFI; median value of fluorescence intensity distribution) expressed as MFI ratio (MFI of antibody-labelled cells/MFI of negative controls)^39^. Gating boundaries were set up using IgG isotype- and fluorochrome-matched and fluorescence minus one (FMO) controls.

### CII recall test

Antigen-specific proliferation of B cells and cytokine production by T cells were examined in dLN cell cultures (3 × 10^5^ cells per well in U-bottomed 96-well plate, Corning, NY, USA). The cells were cultured in the culture medium for 72 h at 37 °C, in 95% air–5% CO_2_ atmosphere, in the presence or in the absence of 5 μg/ml of CII (Sigma-Aldrich Chemie GmbH).

### ELISA

For measuring IL-17 (BioLegend, San Diego, CA, USA), IL-4 (Thermo Fisher Scientific, Waltham, MA, USA) and IFN-γ (R&D Systems, Minneapolis, MN, USA) levels in supernatants of dLN cell cultures, commercial ELISA kits were used with the limits of detection at 8 pg/ml, 2 pg/ml, and less than 10 pg/ml, respectively. All procedures were performed according to the manufacturers’ instructions.

The serum levels of anti-CII IgG antibodies were detected by ELISA as described earlier^9^ with some modifications. Briefly, dilutions of sera (1:100–1:1600 for total IgG, and 1:100 for IgG1, IgG2a, IgG2b) were assayed in 96-well plates (MaxiSorp, Nunc) coated with 5 μg/ml of CII in 50 mM carbonate buffer pH 9.6 and blocked with 2% BSA. Biotin-conjugated secondary antibodies (1:1000; anti-rat IgG, IgG1, IgG2a and IgG2b antibodies, Biolegend Inc., San Diego, CA, USA), streptavidin peroxidase (1:3000) and extrAvidin-peroxidase/o-phenylendiamine system (Sigma, Steinheim, Germany) were used for the detection of specific antibodies. The absorbance was read at 492/620 nm (A_492/620_) on Multiscan Ascent (Labsystems, Helsinki, Finland).

### RT-qPCR

RNA was isolated from dLN tissue or MACS-sorted CD45RA+ cell samples using TRIzol reagent obtained from Thermo Fisher Scientific (Waltham, MA, USA). RNA yield and purity were determined using Orion AquaMate 8000 spectrophotometer (Thermo Scientific, Waltham, MA, USA). cDNA was synthesized from total RNA using High Capacity cDNA Reverse Transcription Kit (Applied Biosystems, Foster City, CA, USA), following the manufacturer’s instructions. RT-qPCR reactions were set up in triplicate (25 μl final volume) using TaqMan Gene Expression Master Mix and premade TaqMan Gene Expression Assays, according to supplier’s protocols (Applied Biosystems). Both reverse transcription and RT-qPCR were performed using Applied Biosystems 7500 Real-Time PCR System, as previously described in detail^10^. TaqMan Gene Expression Assays used in the study: IL-2 (Il2; Rn00587673_m1), IL-21 (Il21; Rn01755623_m1), IL-7 (Il7; Rn00681900_m1), IL-27-subunit p28 (Il27; Rn01510484_m1), T-bet (Tbx21; Rn01461633_m1) and β-actin (Actb; Rn00667869_m1). SDS v1.4.0. software (Applied Biosystems) was used for data analysis. Relative target mRNA expression levels normalized to the internal standard (β-actin) were calculated using the comparative threshold cycle (Ct) method and presented as 2^−dCt^. dCt values were obtained by subtracting Ct values for the internal control gene from Ct values for target genes.

### Statistical analysis

Data were analysed using GraphPad Prism 6 software (GraphPad Software, Inc., La Jolla, CA, USA). Statistically significant differences between the groups were assessed by two-tailed Student’s *t*-test unless otherwise indicated. Data are presented as mean ± SEM. Values of p < 0.05 were considered significant.

## Results

### Greater B cell response to CII in dLNs from female compared with male CIA rats

**Figure 1:**
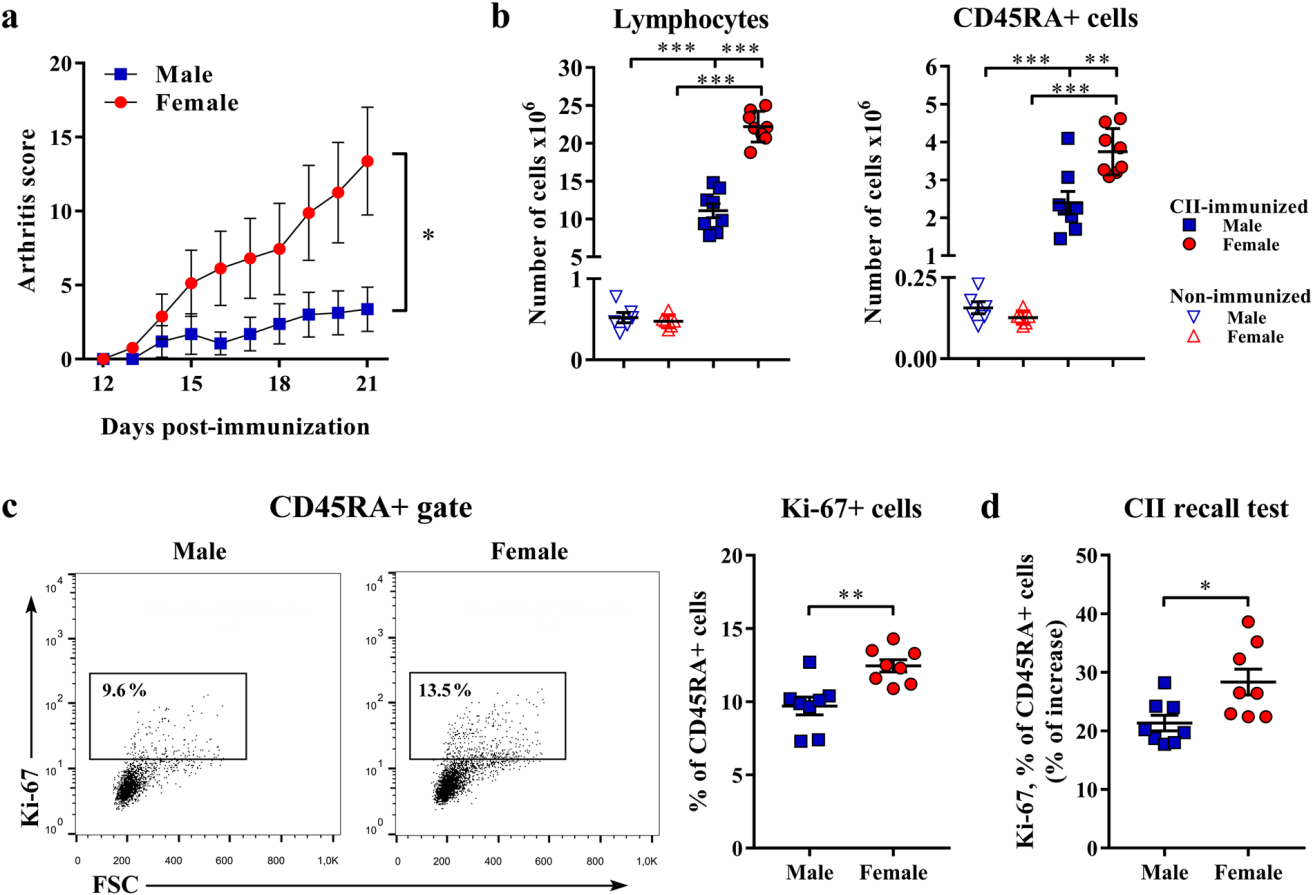
Sex difference in the proliferative capacity of B cells from CIA rats. (**a**) Line graph indicates daily arthritis score in male and female rats until the peak of the disease (on the 21^st^ day post-immunization). Clinical signs were graded on an arbitrary scale as described in Materials and Methods. (**b**) Cells were obtained from lymph nodes draining inflamed joints and nearby tissues (dLNs) recovered at the peak of the clinical severity of CIA. Scatter plots show the absolute number of dLN lymphocytes and the absolute number of B cells (CD45RA+) from CII-immunized and non-immunized male and female rats. Two-way ANOVA (sex × immunization) showed: a significant interaction between factors (F_(1,24)_ = 64.91, p < 0.001) and a significant main effects of sex (F_(1,24)_ = 63.98, p < 0.001) and immunization (F_(1,24)_ = 545.7, p < 0.001) for absolute number of dLN lymphocytes; and a significant interaction between factors (F_(1,24)_ = 11.26, p < 0.01) and a significant main effects of sex (F_(1,24)_ = 10.34, p < 0.01) and immunization (F_(1,24)_ = 183.9, p < 0.001) for absolute number of B cells. Multiplicity corrected p values are reported for Bonferroni tests. (**c**) Representative flow cytometry dot plots show Ki-67 staining of CD45RA+ cells from dLNs of male and female rats. The number indicates the percentage in the region. A scatter plot shows the frequency of proliferating Ki-67+ cells among CD45RA+ cells. (**d**) Scatter plot shows the percentage of increase in the frequency of proliferating Ki-67+ cells among CD45RA+ cells from male and female CII-supplemented dLN cell cultures over CII-free dLN cell cultures from sex-matched rats (CII recall test). Data are presented as means ± SEM. Horizontal lines within scatter plots indicate mean values. n = 6-8 rats per group. *p < 0.05, **p < 0.01, ***p < 0.001.

As previously shown^9,10^, female DA rats develop more severe CIA (Fig. 1a). To test the contribution of sex differences in B-cell antibody response to this dimorphism, B cells recovered from dLNs on the 21^st^ d.p.i. were examined for number and phenotypic properties. Irrespective of sex, more mononuclear cells were recovered from dLNs from CII-immunized rats compared with sex-matched controls, but this increase was more prominent (p < 0.001) in female rats (Fig. 1b). Consequently, differently from popliteal LNs from non-immunized rats exhibiting a similar number of mononuclear cells, more (p < 0.001) mononuclear cells were retrieved from dLNs of CII-immunized female rats compared with their male counterparts (Fig. 1b). Additionally, irrespective of sex, more (p < 0.001) CD45RA+ cells (B cells) were recovered from dLNs of CII-immunized rats when compared with sex-matched non-immunized rats. However, this effect of immunization was more prominent in females (Fig. 1b). Given that their number was comparable between female and male non-immunized rats, more (p < 0.01) CD45RA+ cells were retrieved from dLNs of CII-immunized female rats compared with their male counterparts (Fig. 1b).

Next, the frequency of Ki-67+ activated/proliferating cells among B cells was examined. In accordance with the previous findings, their frequency was higher (p < 0.01) in female rats compared with their male counterparts (Fig. 1c). Given that these findings only indicated that after immunization with CII there were more activated/proliferating cells among female than male rat dLN B cells, but did not provide information about their antigen specificity, dLN B cells were restimulated with CII *in vitro* and the percentage of increase in the frequency of Ki-67+ cells among restimulated B cells over that among non-stimulated B cells (in control cultures without CII) was examined. As expected, upon stimulation with CII greater (p < 0.05) increase in the frequency of Ki-67+ cells was registered among dLN B cells from female rats compared with male ones (Fig. 1d).

**Figure 2:**
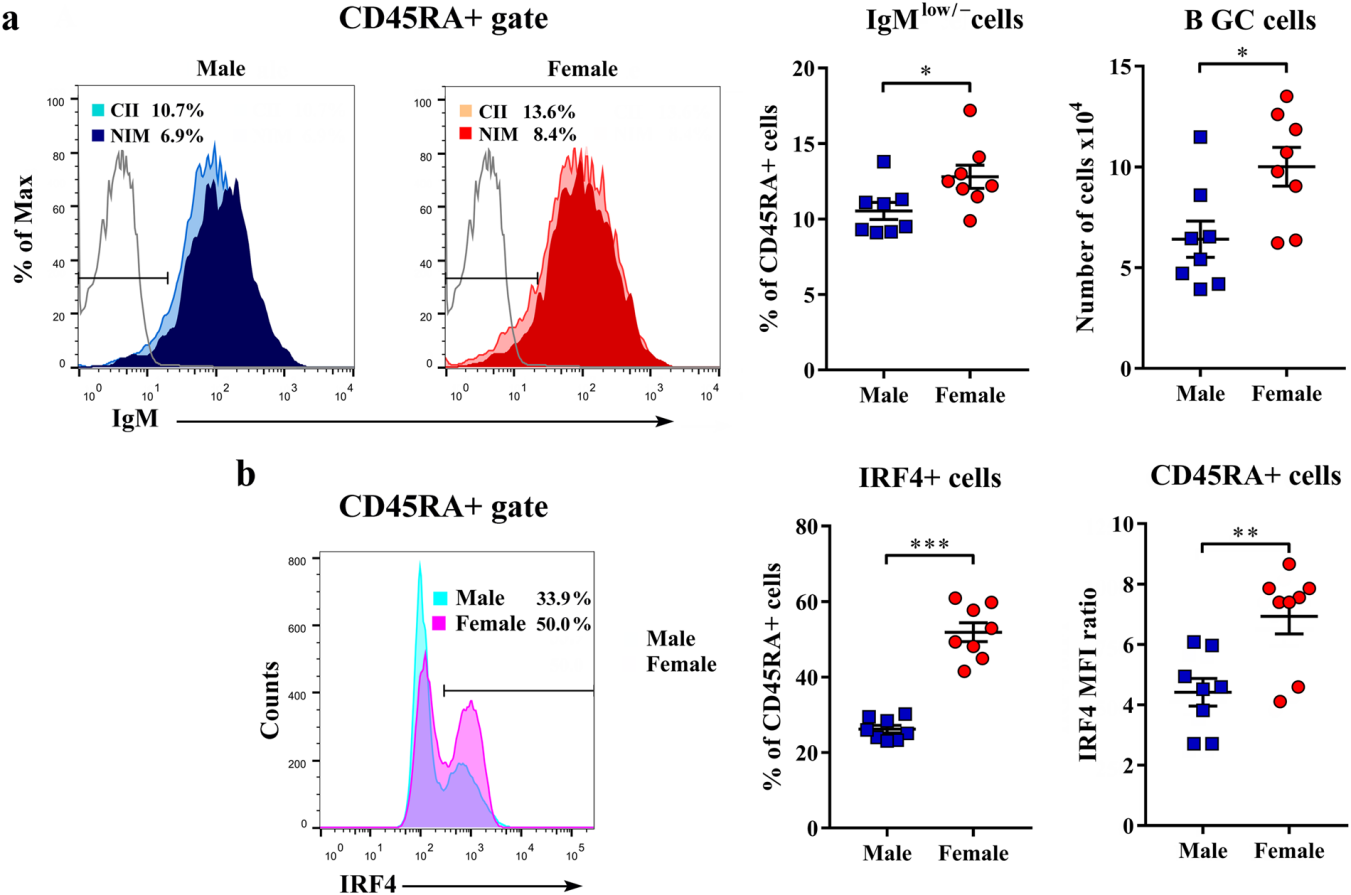
Sex differences in the generation of germinal centre (GC) B cells and IRF4 expression in B cells from CIA rats. Cells were obtained from lymph nodes draining inflamed joints and nearby tissues (dLNs) recovered at the peak of the clinical severity of CIA. (**a**) Representative flow cytometry histogram overlays display IgM expression on CD45RA+ cells from dLNs of male and female CII-immunized (CII) and non-immunized (NIM) rats. The number indicates the percentage in the region. Scatter plots show the frequency of IgM^low/−^ cells among CD45RA+ cells (GC B cells) and the number of GC B cells from dLNs of male and female rats. (**b**) Representative flow cytometry histogram overlay displays IRF4 expression on CD45RA+ cells from dLNs of male and female rats. The number indicates the percentage in the region. Scatter plots show the percentage of IRF4+ cells among CD45RA+ cells and IRF4 mean fluorescence intensity (MFI) ratio of IRF4+ CD45RA+ cells from dLNs of male and female rats. Data are presented as means ± SEM. Horizontal lines within scatter plots indicate mean values. n = 8 rats per group. *p < 0.05, **p < 0.01, ***p < 0.001.

Next, to assess whether the sex difference in *in vitro* activation/proliferation of CII-specific B cells correlated with the sex differences in the frequency of B cells involved in GC reaction (GC B cells), freshly isolated dLN B cells were examined for surface IgM expression. Namely, differently from IgM+ naïve and memory B cells^40^, class-switched IgG antibody-secreting GC B cells are suggested to express IgM^low/−^ phenotype^41^. Indeed, the frequency of IgM^low/−^ cells among dLN B cells, and their number, were greater (p < 0.05) in female than in male rats (Fig. 2a). Next, considering that the transcription factor IRF4 is indispensable not only for GC formation but also for terminal differentiation of B cells to antibody-secreting plasma cells^42^, its expression in dLN B cells was examined. In accordance with the higher frequency of IgM^low/−^ cells among dLN B cells from female rats, the frequency of IRF4+ cells among dLN B cells (p < 0.001), and IRF4 expression level (as indicated by MFI ratio) in these cells (p < 0.01) were greater in female than in male rats (Fig. 2b).

### B cells from dLNs of female CIA rats “enjoy” greater CD4+ T-cell help

**Figure 3:**
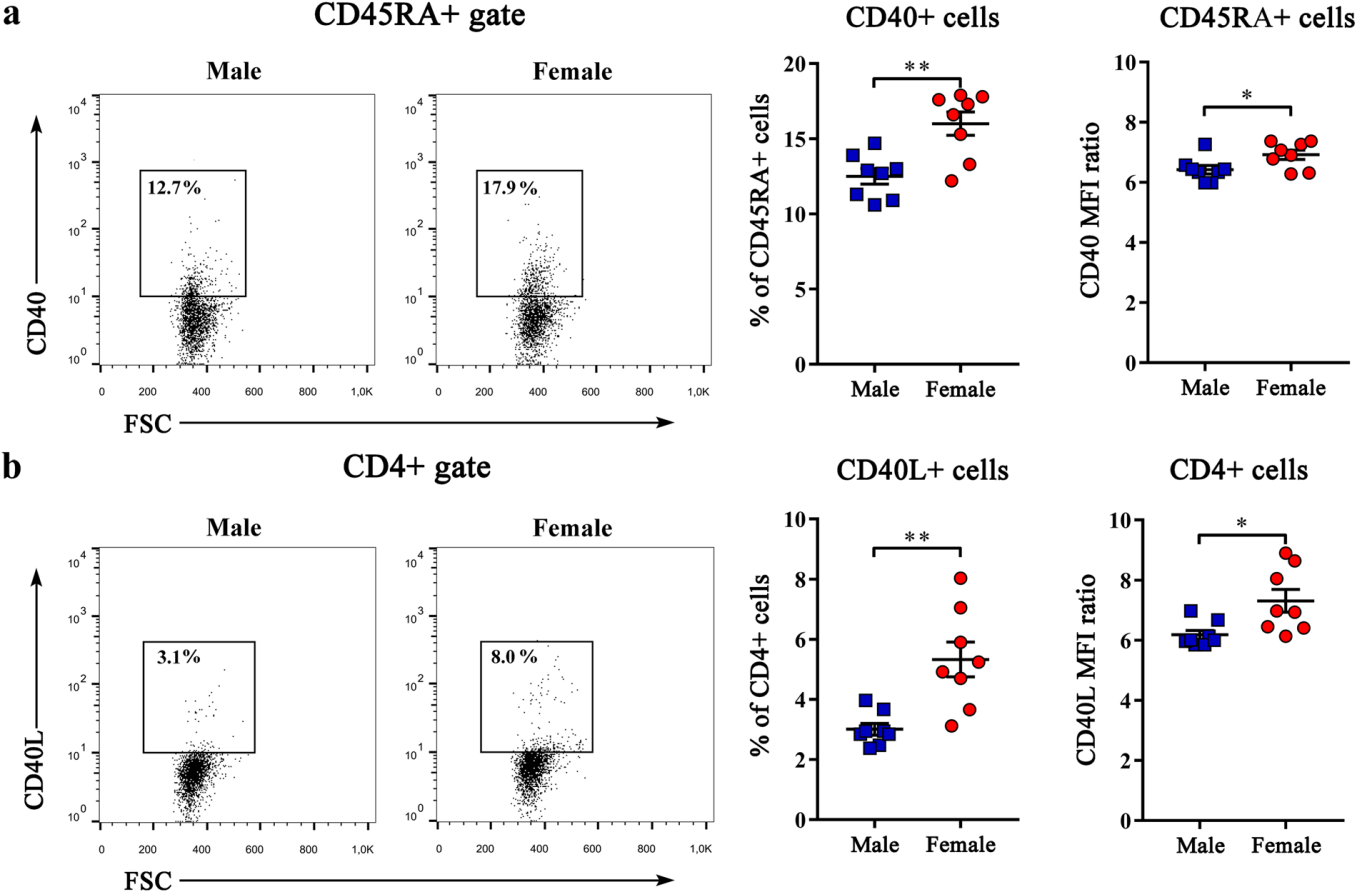
Sex differences in the expression of co-stimulatory molecules CD40 and CD40L (CD154) on lymphocytes from CIA rats. Cells were obtained from lymph nodes draining inflamed joints and nearby tissues (dLNs) recovered at the peak of the clinical severity of CIA. (**a**) Representative flow cytometry dot plots show CD40 staining of CD45RA+ cells from dLNs of male and female rats. The number indicates the percentage in the region. Scatter plots show the frequency of CD40+ cells among CD45RA+ cells and CD40 mean fluorescence intensity (MFI) ratio of CD40+ CD45RA+ cells from dLNs of male and female rats. (**b**) Representative flow cytometry dot plots show CD40L (CD154) staining of CD4+ cells from dLNs of male and female rats. The number indicates the percentage in the region. Scatter plots show the frequency of CD40L + cells among CD4+ cells and CD40L mean fluorescence intensity (MFI) ratio of CD40L+ CD4+ cells from dLNs of male and female rats. Data are presented as means ± SEM. Horizontal lines within scatter plots indicate mean values. n = 8 rats per group. *p < 0.05, **p < 0.01.

Given that GC formation and the production of class-switched antibodies is reliant on the activation of antigen-specific B cells by CD4+ T cells capable of recognizing epitopes of the same antigenic complex, and that CD40:CD40L (CD154) contact is critical in this interaction^43^, the expression of CD40 and CD40L was investigated on CD45RA+ and CD4+ cells recovered from dLNs, respectively. The frequency of CD40+ cells among B cells (Fig. 3a), as well as that of CD40L+ cells among CD4+ cells (Fig. 3b), were higher (p < 0.01) in female than in male rats. Besides, the surface density of CD40 on B cells (Fig. 3a; Supplementary Fig. S1), as that of CD40L on CD4+ cells (Fig. 3b, Supplementary Fig. S1), judging by MFI in the corresponding channels, was greater (p < 0.05) in female than in male rats.

**Figure 4:**
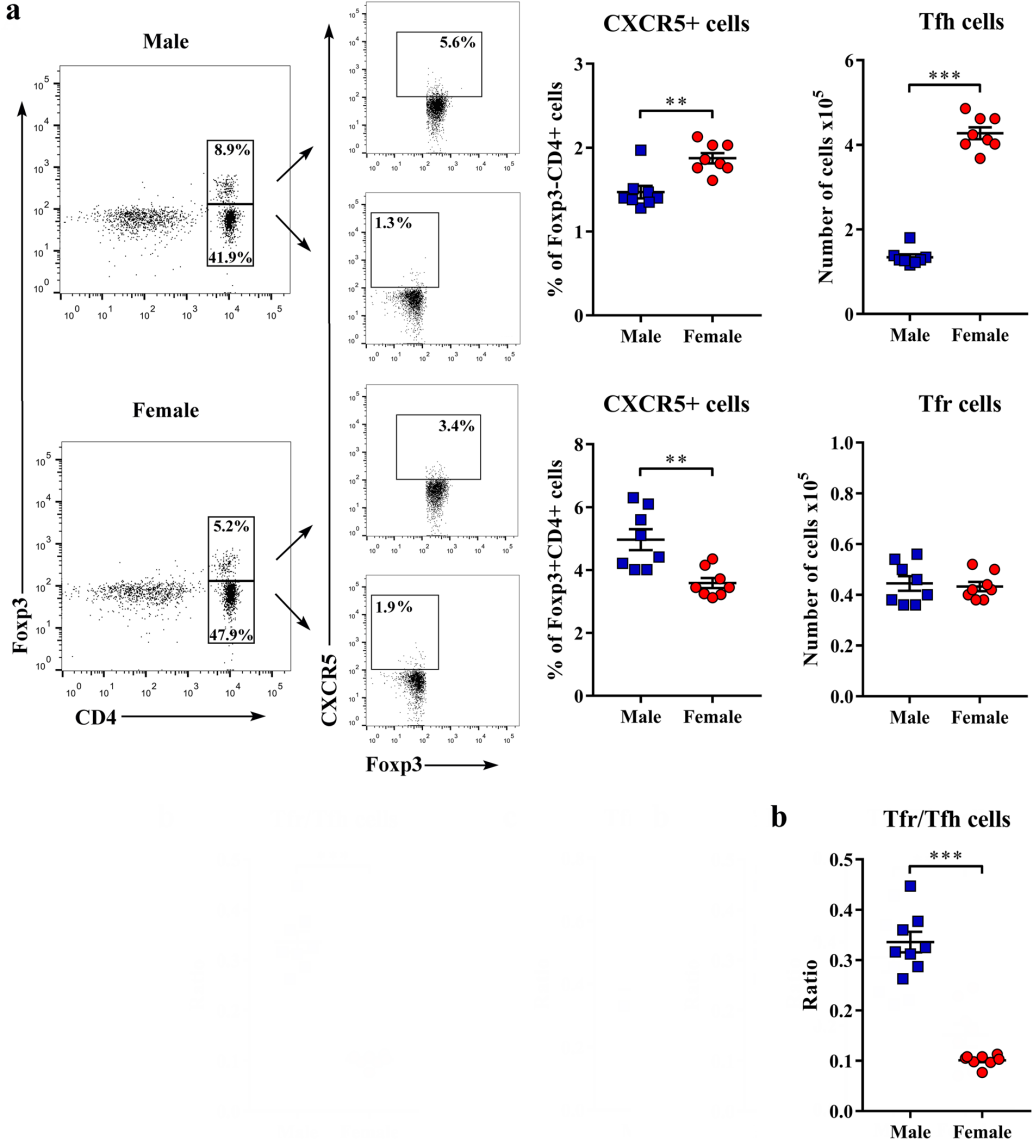
Sex differences in the generation of Tfh cells and Tfr cells in CIA rats. Cells were obtained from lymph nodes draining inflamed joints and nearby tissues (dLNs) recovered at the peak of the clinical severity of CIA. (**a**) Representative flow cytometry dot plots show Foxp3 staining of CD4+ cells and CXCR5 expression on Foxp3+ CD4+ and Foxp3- CD4+ cells from dLNs of male and female rats. The number indicates the percentage in the region. Scatter plots show the frequency of CXCR5+ cells among Foxp3- CD4+ and Foxp3+ CD4+ cells and the absolute number of Tfh and Tfr cells from dLNs of male and female rats. (**b**) A scatter plot shows Tfr/Tfh cell ratio in dLNs of male and female rats. Data are presented as means ± SEM. Horizontal lines within scatter plots indicate mean values. n = 8 rats per group. **p < 0.01, ***p < 0.001.

Considering that Tfh cells specialized in providing help to B cells are essential for GC formation, affinity maturation, and the development of memory B cells^27^, whereas Tfr cells are critical for controlling Tfh cell:B cell interaction^29,30^, dLN cells were also examined for their frequency. To identify these cells, conventional Foxp3-CD4+ cells and Foxp3+ CD4+ cells encompassing predominantly regulatory T cells^44^ (Supplementary Fig. S2) were examined for CXCR5 expression^31^. The frequency of CXCR5+ cells among Foxp3- CD4+ cells (presumably Tfh cells) (p < 0.01) and their number (p < 0.001) were greater in female than in male rat dLNs (Fig. 4a). On the other hand, the frequency of CXCR5+ cells among Foxp3+ CD4+ cells (mainly Tfr cells) was lower (p < 0.01) in female than in male rat dLNs (Fig. 4a). The number of Foxp3+ CD4+ cells was greater (p < 0.05) in females (Supplementary Fig. S2), while the number of Tfr cells was comparable between sexes (Fig. 4a), suggesting their less efficient generation in females. Consequently, Tfr/Tfh cell ratio was shifted (p < 0.001) towards Tfh cells in female compared with male rats (Fig. 4b). The lower percentage of Tfr cells in female than in male rats is suggestive of their impaired differentiation from the precursor cells (Supplementary Fig. S2). It is noteworthy that in non-immunized rats, there were no significant sex differences in the frequency of either Tfh (1.24 ± 0.09% of Foxp3-CD4+ cells in males vs 1.11 ± 0.12% in females) or Tfr (3.2 ± 0.28% of Foxp3+ CD4+ cells in males vs 3.0 ± 0.39% in females) cells in LNs. Thus, it may be assumed that the sex differences in the frequency and the number of Tfh and Tfr cells appeared in the response to immunization.

Given that Tfr cells, apart from Tfh-cell activity, control GC B-cell activity^31,32^, Tfr/B cell ratio in dLNs was also calculated. This ratio was shifted (p < 0.01) towards GC B cells in female rats compared with their male counterparts (Supplementary Fig. S3).

**Figure 5:**
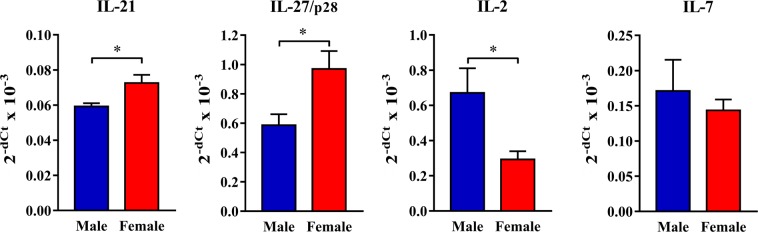
Sex differences in the expression of cytokines regulating the generation of Tfh cells and Tfr cells in CIA rats. Lymph nodes draining inflamed joints and nearby tissues (dLNs) were recovered at the peak of the clinical severity of CIA. Bar graphs show expression of mRNAs for IL-21, IL-27/p28, IL-2 and IL-7 in dLN tissue of male and female rats, as determined by RT-qPCR. Results are represented as 2^−dCt^ relative to β-actin. Data are presented as means ± SEM. n = 8 rats per group. *p < 0.05.

Considering that the differentiation of Tfh cells depends on the balance of cytokines exerting the opposing action on this process^45^, the expression levels of mRNAs for cytokines promoting (IL-21 and IL-27) and inhibiting (IL-2 and IL-7) their differentiation were analysed. The expression levels of mRNAs for IL-21 and IL-27 were greater (p < 0.05), whereas that of IL-2 mRNA was less (p < 0.05) in female than in male rat dLNs (Fig. 5). On the other hand, a similar amount of mRNA encoding IL-7 was found in female and male dLNs (Fig. 5).

### Female CIA-affected rats mount higher total serum level of CII-specific antibodies and exhibit the shift in IgG2a(b)/IgG1 ratio towards an IgG2a(b) response

**Figure 6:**
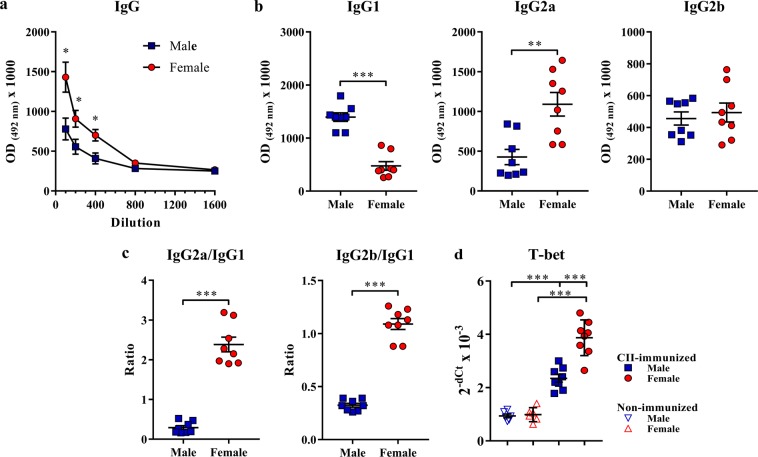
Sex differences in serum levels of CII-specific IgG antibodies and T-bet mRNA expression in B cells in CIA rats. Serum was obtained at the peak of the clinical severity of CIA. (**a**) Line graph indicates the serum levels of anti-CII IgG in male and female rats, as shown by mean optical densities (OD) at 492 nm in ELISA anti-IgG reaction performed with increasing dilutions of the sera. (**b**) Scatter plots represent serum (1:100 dilution) levels (OD at 492 nm) of IgG1, IgG2a and IgG2b antibodies. (**c**) Scatter plots represent IgG2a/IgG1 and IgG2b/IgG1 antibody level ratios in male and female rats. (**d**) Scatter plot shows mRNA expression of T-bet in B cells purified from lymph nodes draining inflamed joints and nearby tissues (dLNs) from CII-immunized (at the peak of clinical severity of CIA) and from non-immunized male and female rats. Two-way ANOVA (sex x immunization) showed: a significant interaction between factors (F_(1,24)_ = 18.41, p < 0.001) and a significant main effects of sex (F_(1,24)_ = 20.88, p < 0.001) and immunization (F_(1,24)_ = 156.2, p < 0.001). Multiplicity corrected p values are reported for Bonferroni tests. Data are presented as means ± SEM. Horizontal lines within scatter plots indicate mean values. n = 6–8 rats per group. **p < 0.01, ***p < 0.001.

To examine the significance of GC reaction for the sex differences in the production of autoantibodies in response to immunization with CII, the total serum level of CII-specific IgG and its subclasses were examined. The serum level of anti-CII IgG antibody was greater (p < 0.05) in female than in male rats (Fig. 6a). Considering sex differences in the IgG autoantibody subclass profile in some other autoimmune diseases^46^, the subclass profile of IgG specific to CII was additionally assessed. The serum level of CII-specific IgG1 antibody^47,48^ was lower (p < 0.001) in female rats than in their male counterparts (Fig. 6b). On the contrary, the serum level of CII-specific IgG2a antibody shown to be highly pathogenic^47,48^ was higher (p < 0.01) in female rats than in their male counterparts (Fig. 6b). There was no sex difference in the serum level of CII-specific IgG2b antibody (Fig. 6b). Consequently, compared with male rats, in female rats IgG2a/IgG1 and IgG2b/IgG1 antibody ratios were shifted (p < 0.001) towards IgG2a and IgG2b antibody, respectively (Fig. 6c). Given that T-bet promotes class switching to IgG2a^49^, the expression level of mRNA for T-bet was investigated in B cells recovered from dLNs of CII-immunized rats of both sexes, and in B cells isolated from the popliteal LNs from non-immunized control rats. There was no sex difference in T-bet mRNA expression in B cells from non-immunized rats. However, following immunization with CII greater increase (p < 0.001) in T-bet mRNA expression was observed in female than in male rat dLN B cells (Fig. 6d).

### Sex differences in CII-specific IgG subclass profile correlate with those in T-cell cytokine profile

**Figure 7:**
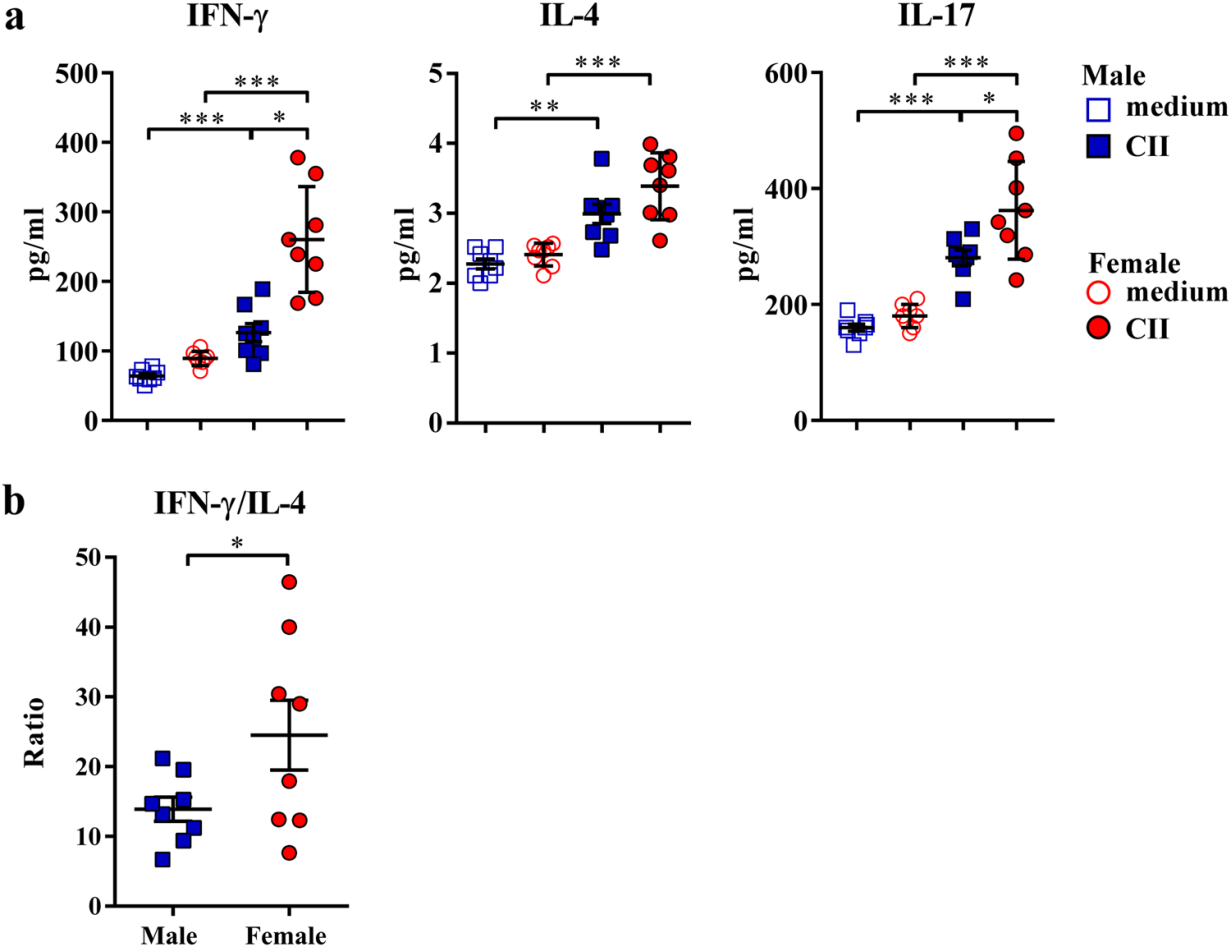
Sex differences in the capacity of T cells to produce Th1, Th2 and Th17 signature cytokines in CII-stimulated cell cultures. Cultured cells were obtained from lymph nodes draining inflamed joints and nearby tissues (dLNs) recovered at the peak of clinical severity of CIA. (**a**) Scatter plots indicate the concentration of IFN-γ, IL-4 and IL-17 in 72-hour dLN cell cultures from male and female rats without CII (medium) or with CII (CII). Two-way ANOVA (sex x culturing condition) showed: a significant interaction between factors (F_(1,28)_ = 13.04, p < 0.01) and a significant main effects of sex (F_(1,28)_ = 27.84, p < 0.001) and culturing conditions (F_(1,28)_ = 59.91, p < 0.001) for IFN-γ; a significant main effect of culturing conditions (F_(1,28)_ = 49.34, p < 0.001) for IL-4; and a significant main effects of sex (F_(1,28)_ = 9.06, p < 0.01) and culturing conditions (F_(1,28)_ = 80.86, p < 0.001) for IL-17. (**b**) Scatter plot shows IFN-γ/IL-4 concentration ratio in 72-hour dLN cell cultures with CII from male and female rats. Data are presented as means ± SEM. Horizontal lines within scatter plots indicate mean values. n = 8 rats per group. *p < 0.05, **p < 0.01, ***p < 0.001.

Considering that although IFN-γ and IL-4 predominantly regulate IgG class switching in rats^22,23^, IL-17 could also contribute to its shaping, as IL-17 deficient mice exhibit impaired production of collagen-specific IgG2a antibodies^24^, dLNs were examined for the frequency of IFN-γ-, IL-4- and IL-17-producing T cells. The frequencies of T cells producing IFN-γ and IL-17, the cytokines favouring production of IgG2 antibody^22,24^, were higher (p < 0.05) in female than in male rat dLNs, whereas there was no sex difference in the frequency of IL-4+ T cells (Supplementary Fig. S4). Additionally, the concentrations of IFN-γ, IL-17, and IL-4 were examined in CII-stimulated dLN cell cultures. There were no sex differences in the production of these cytokines in control (medium only) cultures (Fig. 7a). Their concentrations increased in CII-stimulated dLN cell cultures from rats of both sexes when compared with sex-matched control cultures (Fig. 7a). The concentrations of IFN-γ and IL-17 were greater (p < 0.05) in female than in male rat CII-stimulated dLN cell cultures (Fig. 7a). On the other hand, IL-4 concentration was comparable in CII-stimulated dLN cell cultures from female and male rats (Fig. 7a). Considering that IFN-γ/IL-4 production level ratio rather than the production level of any of these two cytokines is important in regulating IgG class switching^50^, IFN-γ/IL-4 production ratio was calculated. Indeed, consistent with IgG2a/IgG1 antibody ratio, this ratio was shifted (p < 0.05) towards IFN-γ in female rat CII-stimulated dLN cell cultures compared with corresponding cultures from male rats (Fig. 7b).

## Discussion

The present study extended our earlier findings on sexual dimorphism in susceptibility to CIA^9,10^ recapitulating female predominance observed in RA by showing that female rats mount quantitatively greater and qualitatively different CII-specific IgG response compared with their male counterparts. This was associated with the more efficient generation of Tfh cells and most likely CD40L/CD40-mediated interactions between Th cells and B cells, and the shift in Th1/Th2 signature cytokine production ratio towards the former in females compared with males. The greater total serum CII-specific IgG response in female rats compared with male counterparts was consistent with data indicating that, generally, females mount a stronger IgG response than males^36,37^. Additionally, this was in accordance with data indicating that greater severity of rheumatoid arthritis in women than man could be ascribed to the stronger humoral immune response in women^51^. However, the analysis of the influence of sex on the antibody of distinct specificities showed it may vary depending on antibody specificity^52,53^. Furthermore, differently from rat CIA models and the human disease, in several mouse CIA models sex differences in serum levels of autoantibodies have not been found^54,55^, suggesting that this dimorphism may be not only antigen but also species/strain specific. The stronger total IgG response in female compared with male CIA-affected rats correlated with the greater number of GC B cells in dLNs from female rats. Of note, this reflected a more prominent increase in their number in the response to immunization with CII in dLNs from female compared with male rats. This correlated with the greater frequency of activated/proliferating Ki-67+ cells among dLN B cells from female rats compared with male rats. To the best of our knowledge, there is no data on sex differences in intrinsic B-cell activation/proliferation capacity. In a model of RA induced by transfer of hematopoietic stem cells from RA patients into immunodeficient mice, more B cells were retrieved from peripheral blood of female recipients compared with male recipients, indicating that sex-specific extrinsic microenvironmental factors rather than B-cell intrinsic factors contributed to the sex differences in the B-cell number^56^. Additionally, it was shown that estrogen increases the number of autoreactive B cells in the naive repertoire by protecting/increasing the survival of the immature B cells that would normally be deleted^57^. The greater proliferation of B cells in CII-stimulated dLN cell cultures from female compared with male rats is fully consistent with this finding. Furthermore, it was found that estrogen acting directly on B cells upregulates not only genes involved in B-cell survival but also in their activation^57^. These findings have been associated with an increased propensity towards the development of autoimmune diseases in female compared with male rats^57^. In the same vein, androgens were shown to have the opposite effect on B-cell survival providing an additional explanation for sex differences in susceptibility to antibody-mediated autoimmune diseases^58^. Consistently, we found a lower frequency of Annexin V+ cells among B cells from female rats compared with their male counterparts (Supplementary Fig. S5).

On the other hand, considering that Tfh cells provide critical signals to antigen-primed B cells in GCs to undergo proliferation during an immune response^27^, the greater activation/proliferation of B cells from female rats could be associated not only with the greater number of CII-specific B cells, but also with the more Tfh cells in female compared with male rat dLNs. This may be linked with estrogen-sensitive PPARγ/dependent differentiation of Tfh cells^59^. A more prominent increase in the number of Tfh cells in female rat dLNs following immunization with CII leading to sex bias in their number in immunized rats (greater number in females) was consistent with data indicating that Th cells from adult women and female mice have greater proliferative capacity than those from age-matched men and male mice, respectively^60,61^. Additionally, we have recently shown the greater activation/proliferation of Th cells from dLNs of female CII-immunized DA rats compared with their male counterparts^10^. Our findings also revealed the enhanced expression of IL-21 and IL-27, the cytokines supporting Tfh-cell differentiation^45^, in female compared with male rat dLN cells. Given that IL-12p/35 stimulates IL-21 expression^45^, the upregulated expression of IL-21 in dLN cells from females compared with males was consistent with the greater IL-12p/35 expression in their dLNs^10^. It should be emphasized that IL-21, as IL-27^62^, supports not only Tfh-cell differentiation and thereby GC B-cell formation and function, but also directly acts on B cells supporting their growth, survival, and antibody secretion^33^. It is noteworthy that in RA patients the increased serum IL-21 levels correlate with the disease activity, frequency of Tfh-like cells and anti-citrullinated peptide antibody level^33^. To the greater Tfh-cell generation in female than in male rats' dLNs could also contribute the lower expression of IL-2, the cytokine involved in the negative regulation of Tfh-cell differentiation^45^, in female rat dLN cells. In favour of our findings are data indicating that estrogen-stimulated CD40L expression on activated CD4+ T cells contributes to a greater incidence of several antibody-mediated autoimmune diseases in women^63,64^. To point out to the significance of this finding are data indicating that: i) the increased expression of CD40L on peripheral blood CD4+ T cells has been approved as a marker of disease activity in RA^65^ and ii) the CD40L/CD40 pathway has recently been recognized as a potential target for innovative therapy in several autoimmune diseases, including RA^42^. Additionally, greater expression of CD40 was found in B cells from women compared with men^66^. This dichotomy was associated with the greater circulating 17β-estradiol level in women and suggested to be critical for sex-based differences in autoimmune disease incidence^67^.

Given that Tfh/Tfr cell ratio rather than solely the number of Tfh cells determines the magnitude of antibody response^31,43^, it should be emphasized that Tfr/Tfh cell ratio was shifted towards Tfh cells in female compared with male rat dLNs. To corroborate this finding, compared with male mice in female mice mounting stronger antibody response to influenza vaccine antigens the shift in Tfr/Tfh cell ratio to the Tfh cell side in secondary lymph organs was also found^50^. Additionally, lack of Tfr cells or the shift Tfr/Tfh cell ratio towards Tfh cells is shown to increase the risk of autoimmunity and autoantibody^68^. Considering the critical role of a CD40L/CD40-mediated interactions in the establishment of an effective thymus-dependent humoral immune response and pathogenesis of experimental autoimmune diseases^69^, including CIA^70^, the greater antibody response in female compared with male CIA rats could be partly ascribed to the greater density of CD40L and CD40 on CD4+ T cells and B cells, respectively.

Further, given that Tfr cells shape the GC response and balance tolerance not only through interactions with Tfh (by modifying their number and function, and thereby GC B-cell response), but also directly interacting with B cells^71^, the lower Tfr/GC B-cell ratio in female compared with male rat dLNs could also contribute to the greater CII-specific IgG antibody response in females, and consequently the greater severity of the disease.

Finally, the greater magnitude of CII-specific IgG response in females compared with males could be linked with the greater expression of IRF4 in B cells, as its enhanced expression upregulates the expression of B lymphocyte-induced maturation protein 1 (Blimp1) in B cells, and consequently their differentiation into antibody-secreting plasma cells^42^. Thus, the higher frequency of IRF4+ cell in female rat B cells indicates more plasma cells in their dLNs^42^. To additionally support this notion it should be added that IRF4 was identified as a particularly important protein involved in the RA-mediated production of IgG in stimulated B cells^72^. Sex difference in the expression of IRF4 could also be linked with findings indicating that estrogen upregulates IRF4 expression in dendritic cells^73^.

Moreover, it should be emphasized that sex differences were also found in qualitative characteristics of IgG response, as the serum CII-specific IgG2a(b)/IgG1 ratio was shifted towards an IgG2a(b) response in females compared with males, reflecting reduced levels of IgG1 accompanied by increased and unaltered levels of IgG2a and IgG2b, respectively. The higher serum level of IgG2a could be related to the greater expression of T-bet in B cells from female rats affected by CIA compared with their male counterparts, as it was shown T-bet has a crucial role in the switching antibody response toward IgG2a^74^. In favour of these findings was the sexual dimorphism in serum IgG subclass profile in response to stimulation with some other (auto)antigens in humans and mice^46,50^. The shift in IgG2a/IgG1 ratio in females compared with males was in accordance with the higher frequency of INF-γ-producing T cells in dLNs from female compared with male rats, and the higher INF-γ levels in CII-stimulated female rat dLN cell cultures^22^ leading to the shift in INF-γ/IL-4 production ratio to the side of INF-γ. This shift in INF-γ/IL-4 production ratio in female CIA-affected rats could be ascribed to the greater expression of T-bet in their B cells^74^. To corroborate this finding are data from some previous studies indicating disease-associated sexual dimorphism in the production levels of Th1/Th2 signature cytokines, i.e. so-called “cytokine level network profile”^75^. To additionally corroborate the higher serum level of IgG2a antibody was the higher frequency of IL-17+ T cells in dLNs from female compared with male rats and IL-17 level in CII-stimulated dLN cell cultures from female rats^24^, as well as enhanced expression of IL-27 in female rat dLNs^76^. On the other hand, despite the lower serum level of IgG1 in females, the frequency of T cells producing IL-4, the cytokine associated with regulation of IgG1 antibody production^23^, in dLNs was comparable between sexes, whereas IL-4 levels in CII-stimulated dLN cell cultures did not significantly differ between female and male rats. This was in line with previous data indicating that the ratio between production levels of cytokines driving IgG subclass switching rather than their absolute concentrations determines IgG subclass profile^50^. Additionally, given that Tfr cells acting on GC B cells either directly or indirectly, via Tfh-mediated mechanism, influence IgG switching^77^, it may be speculated that sex differences in Tfr-cell regulatory action also contributed to the sex bias in IgG subclass profile. The relationship between the serum levels of different CII-specific IgG subclasses and clinical CIA is not well-defined. Effective therapies of CIA in rats have been associated with the decline in IgG2a^48,78^ and IgG2b^47,48^ antibody levels and unaltered^47,48,79^/increased^47^ levels of IgG1, suggesting the relevance of IgG2/IgG1 level ratio as a parameter of the severity of the disease. This could be ascribed to differences in functional properties of distinct IgG subclasses (isotypes)^80^. In this context, it should be pointed out that CII-specific IgG2a antibody, as the most potent activator of Fc receptor-bearing macrophages among IgG antibodies^80^, is suggested to have a key pathogenic role in rats^47,48^ and mice^81,82^. However, it should be also mentioned that there are data indicating that pathogenic potential of district IgG subclasses did not substantially differ in some mouse CIA models^54,55^. This inconsistency could be reconciled by data indicating that pathogenic effects of IgG antibody depend not only on the antibody class/subclass profile, but also on their titres and the target organ reflecting the abundance of the target FcγR bearing inflammatory cells^21^. Thus, considering the greater infiltration of CIA-inflicted paws with inflammatory cells^9^, particularly ED1+ macrophages (data not shown), it may be speculated that the greater generation of IgG2a antibody in female rats compared with their male counterparts was of pathogenic significance.

**Figure 8:**
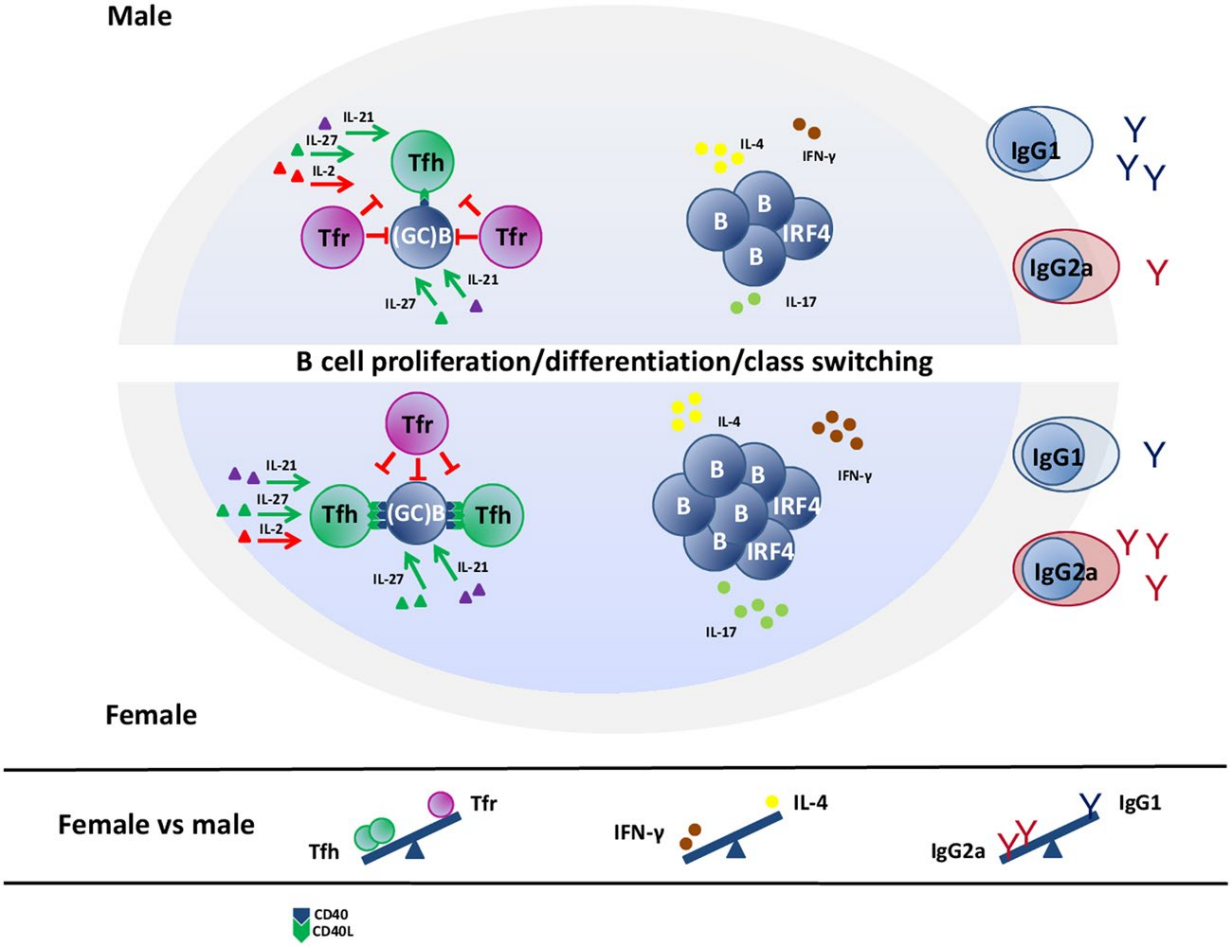
Schematic presentation of mechanisms underlying sex differences in germinal centre (GC) reaction leading to the greater magnitude and more pathogenic profile of IgG response in female compared with male rats immunized with collagen type II (CII). The greater magnitude of the serum CII-specific IgG response in female compared with male rats could be associated with the greater number of GC B cells in lymph nodes draining inflamed joints and adjacent tissue (dLNs) from females reflecting most likely the greater number of CII-specific B cells, their augmented proliferation, and increased IRF4 expression. This was consistent with the greater proliferation of Th cells and a more efficient generation of Tfh cells in female dLNs expressing a more favourable cytokine milieu [mirrored in greater and less expression of cytokines supporting (IL-21 and IL-27) and inhibiting (IL-2) Tfh-cell differentiation, respectively] followed by comparable number of Tfr cells exerting suppressive action on B cells either directly or indirectly acting on Tfh cells. Hence, in female rats, Tfr/Tfh and Tfr/B-cell ratios were shifted towards Tfh and GC B cells, respectively. Additionally, to the greater serum total CII-specific IgG response in female compared with male rats contributed a more efficient T-cell to B-cell communication trough CD40L/CD40 interactions (judging by their surface densities), and increased IL-21/IL-27 expression in dLN cells. The shift in the serum CII-specific IgG2a/IgG1 antibody ratio to the side of IgG2a antibody in female compared with male rats reflected the shifted INF-γ/IL-4 production ratio toward INF-γ, and possibly sex difference in the total Tfr-cell suppressive capacity in female rats. Red arrows (inhibition); green arrows (stimulation).

In conclusion, the current study showed the greater magnitude of serum CII-specific IgG response with the prevalence of IgG2a antibodies in females exhibiting greater susceptibility to CIA, and pointed out to the putative role of T-cell mechanisms in these dimorphisms. Consequently, it suggested the new T-cell targets for further translational pharmacology research (Fig. 8) aimed to identify sex-specific treatments in RA to improve therapeutic success rates.

## Supplementary information

Dataset 1.

## References

[1] Firestein GS, McInnes IB (2017). Immunopathogenesis of rheumatoid arthritis. Immunity.

[2] Forslind K, Hafstrom I, Ahlmen M, Svensson B, Group BS (2007). Sex: a major predictor of remission in early rheumatoid arthritis?. Ann. Rheum. Dis..

[3] Cincinelli G, Generali E, Dudam R, Ravindran V, Selmi C (2018). Why women or why not men? sex and autoimmune diseases. Indian J. Rheumatol..

[4] Ganesan K (2008). Gender differences and protective effects of testosterone in collagen induced arthritis in rats. Rheumatol. Int..

[5] Cutolo M (2007). Sex and rheumatoid arthritis: mouse model versus human disease. Arthritis Rheum..

[6] Luckey D, Medina K, Taneja V (2012). B cells as effectors and regulators of sex-biased arthritis. Autoimmunity.

[7] Holmdahl R (1995). Female preponderance for development of arthritis in rats is influenced by both sex chromosomes and sex steroids. Scand. J. Immunol..

[8] Remmers EF (2002). Modulation of multiple experimental arthritis models by collagen-induced arthritis quantitative trait loci isolated in congenic rat lines: different effects of non-major histocompatibility complex quantitative trait loci in males and females. Arthritis Rheum..

[9] Dimitrijević M (2018). Collagen-induced arthritis in Dark Agouti rats as a model for study of immunological sexual dimorphisms in the human disease. Exp. Mol. Pathol..

[10] Dimitrijević M (2019). Sexual dimorphism in Th17/Treg axis in lymph nodes draining inflamed joints in rats with collagen-induced arthritis. Brain Behav. Immun..

[11] Hill NJ, Hultcrantz M, Sarvetnick N, Flodstrom-Tullberg M (2007). The target tissue in autoimmunity-an influential niche. Eur. J. Immunol..

[12] Klein SL, Flanagan KL (2016). Sex differences in immune responses. Nat. Rev. Immunol..

[13] Ngo ST, Steyn FJ, McCombe PA (2014). Gender differences in autoimmune disease. Front. Neuroendocrinol..

[14] Lamacchia C, Palmer G, Seemayer CA, Talabot-Ayer D, Gabay C (2010). Enhanced Th1 and Th17 responses and arthritis severity in mice with a deficiency of myeloid cell-specific interleukin-1 receptor antagonist. Arthritis Rheum..

[15] Yap Hooi-Yeen, Tee Sabrina, Wong Magdelyn, Chow Sook-Khuan, Peh Suat-Cheng, Teow Sin-Yeang (2018). Pathogenic Role of Immune Cells in Rheumatoid Arthritis: Implications in Clinical Treatment and Biomarker Development. Cells.

[16] Cohen SB (2006). Rituximab for rheumatoid arthritis refractory to anti-tumor necrosis factor therapy: Results of a multicenter, randomized, double-blind, placebo-controlled, phase III trial evaluating primary efficacy and safety at twenty-four weeks. Arthritis Rheum..

[17] Dahdah A (2018). Germinal center B cells are essential for collagen-induced arthritis. Arthritis Rheumatol..

[18] Silverman GJ, Carson DA (2003). Roles of B cells in rheumatoid arthritis. Arthritis Res. Ther..

[19] Svensson L, Jirholt J, Holmdahl R, Jansson L (1998). B cell-deficient mice do not develop type II collagen-induced arthritis (CIA). Clin. Exp. Immunol..

[20] Yanaba K (2007). B cell depletion delays collagen-induced arthritis in mice: arthritis induction requires synergy between humoral and cell-mediated immunity. J. Immunol..

[21] Nandakumar KS (2010). Pathogenic antibody recognition of cartilage. Cell Tissue Res..

[22] Gracie JA, Bradley JA (1996). Interleukin-12 induces interferon-gamma-dependent switching of IgG alloantibody subclass. Eur. J. Immunol..

[23] Saoudi A (1999). Experimental autoimmune myasthenia gravis may occur in the context of a polarized Th1- or Th2-type immune response in rats. J. Immunol..

[24] Nakae S (2003). IL-17 production from activated T cells is required for the spontaneous development of destructive arthritis in mice deficient in IL-1 receptor antagonist. Proc. Natl. Acad. Sci. USA.

[25] Moschovakis GL (2017). T cell specific Cxcr5 deficiency prevents rheumatoid arthritis. Sci. Rep..

[26] Benaglio Francesca, Vitolo Barbara, Scarabelli Martina, Binda Elisa, Bugatti Serena, Caporali Roberto, Montecucco Carlomaurizio, Manzo Antonio (2015). The Draining Lymph Node in Rheumatoid Arthritis: Current Concepts and Research Perspectives. BioMed Research International.

[27] Crotty S (2014). T follicular helper cell differentiation, function, and roles in disease. Immunity.

[28] Maceiras AR, Fonseca VR, Agua-Doce A, Graca L (2017). T follicular regulatory cells in mice and men. Immunology.

[29] Zhu Y, Zou L, Liu YC (2016). T follicular helper cells, T follicular regulatory cells and autoimmunity. Int. Immunol..

[30] Gensous N (2018). T follicular helper cells in autoimmune disorders. Front. Immunol..

[31] Sage PT, Sharpe AH (2015). T follicular regulatory cells in the regulation of B cell responses. Trends Immunol..

[32] Chung Y (2011). Follicular regulatory T cells expressing Foxp3 and Bcl-6 suppress germinal center reactions. Nat. Med..

[33] Liu R (2012). A regulatory effect of IL-21 on T follicular helper-like cell and B cell in rheumatoid arthritis. Arthritis Res. Ther..

[34] Niu Q (2018). Enhanced IL-6/phosphorylated STAT3 signaling is related to the imbalance of circulating T follicular helper/T follicular regulatory cells in patients with rheumatoid arthritis. Arthritis Res. Ther..

[35] Fan H (2014). Gender differences of B cell signature in healthy subjects underlie disparities in incidence and course of SLE related to estrogen. J. Immunol. Res..

[36] Teixeira D (2011). Evaluation of lymphocyte levels in a random sample of 218 elderly individuals from Sao Paulo city. Rev. Bras. Hematol. Hemoter..

[37] Furman D (2014). Systems analysis of sex differences reveals an immunosuppressive role for testosterone in the response to influenza vaccination. Proc. Natl. Acad. Sci. USA.

[38] Hou W (2010). A systematic comparison between collagen-induced arthritis and pristane-induced arthritis in Dark Agouti rats. Clin. Exp. Rheumatol..

[39] Reguzzoni M (2002). Ultrastructural localization of tyrosine hydroxylase in human peripheral blood mononuclear cells: effect of stimulation with phytohaemagglutinin. Cell Tissue Res..

[40] Kil LP (2012). Btk levels set the threshold for B-cell activation and negative selection of autoreactive B cells in mice. Blood.

[41] Shinall SM, Gonzalez-Fernandez M, Noelle RJ, Waldschmidt TJ (2000). Identification of murine germinal center B cell subsets defined by the expression of surface isotypes and differentiation antigens. J. Immunol..

[42] Willis SN (2014). Transcription factor IRF4 regulates germinal center cell formation through a B cell-intrinsic mechanism. J. Immunol..

[43] Karnell Jodi L., Rieder Sadiye Amcaoglu, Ettinger Rachel, Kolbeck Roland (2019). Targeting the CD40-CD40L pathway in autoimmune diseases: Humoral immunity and beyond. Advanced Drug Delivery Reviews.

[44] Sage PT, Tan CL, Freeman GJ, Haigis M, Sharpe AH (2015). Defective TFH cell function and increased TFR cells contribute to defective antibody production in aging. Cell Rep..

[45] Read KA, Powell MD, Oestreich KJ (2016). T follicular helper cell programming by cytokine-mediated events. Immunology.

[46] Wong AH, Agrawal N, Hughes GC (2015). Altered IgG autoantibody levels and CD4(+) T cell subsets in lupus-prone Nba2 mice lacking the nuclear progesterone receptor. Autoimmunity.

[47] Lu S, Holmdahl R (1999). Different therapeutic and bystander effects by intranasal administration of homologous type II and type IX collagens on the collagen-induced arthritis and pristane-induced arthritis in rats. Clin. Immunol..

[48] So JS (2008). Lactobacillus casei potentiates induction of oral tolerance in experimental arthritis. Mol. Immunol..

[49] Peng SL, Szabo SJ, Glimcher LH (2002). T-bet regulates IgG class switching and pathogenic autoantibody production. Proc. Natl. Acad. Sci. USA.

[50] Arsenović-Ranin N (2019). Influence of aging on germinal centre reaction and antibody response to inactivated influenza virus antigens in mice: sex-based differences. Biogerontology.

[51] Straub RH (2007). The complex role of estrogens in inflammation. Endocr Rev..

[52] Pertsinidou E (2019). Ab1285 IgA RF is associated with high age of rheumatoid arthritis onset. Ann. Rheum. Dis..

[53] Jawaheer D, Lum RF, Gregersen PK, Criswell LA (2006). Influence of male sex on disease phenotype in familial rheumatoid arthritis. Arthritis Rheum..

[54] Taneja V (2007). HLA-DR4-transgenic mice that mimic the sex bias of rheumatoid arthritis. Arthritis Rheum..

[55] Campbell, I. K., Hamilton, J. A., Wicks, I. P. Collagen-induced arthritis in C57BL/6 (H-2b) mice: new insights into an important disease model of rheumatoid arthritis. *Eur. J. Immunol*. **30**(6),1568–75, doi:10.1002/1521-4141(200006)30:6<1568::AID-IMMU1568>3.0.CO;2-R (2000). 10.1002/1521-4141(200006)30:6<1568::AID-IMMU1568>3.0.CO;2-R 10898492

[56] Borsotti C (2018). HSC extrinsic sex-related and intrinsic autoimmune disease-related human B-cell variation is recapitulated in humanized mice. Blood Adv..

[57] Grimaldi CM, Cleary J, Dagtas AS, Moussai D, Diamond B (2002). Estrogen alters thresholds for B cell apoptosis and activation. J. Clin. Invest..

[58] Altuwaijri S (2009). Susceptibility to autoimmunity and B cell resistance to apoptosis in mice lacking androgen receptor in B cells. Mol. Endocrinol..

[59] Park HJ, Park HS, Lee JU, Bothwell AL, Choi JM (2016). Gender-specific differences in PPARγ regulation of follicular helper T cell responses with estrogen. Sci. Rep..

[60] Waskowska A (2017). Influence of oxygen concentration on T cell proliferation and susceptibility to apoptosis in healthy men and women. Folia Histochem. Cytobiol..

[61] Rodriguez-Lara V, Muñiz-Rivera Cambas A, González Villalva A, Fortoul TI (2016). Sex-based differences in lymphocyte proliferation in the spleen after vanadium inhalation. J. Immunotox..

[62] Vijayan D (2016). IL-27 directly enhances germinal center B cell activity and potentiates lupus in sanroque mice. J. Immunol..

[63] Rider V (2001). Estrogen increases CD40 ligand expression in T cells from women with systemic lupus erythematosus. J. Rheumatol..

[64] Jobling K, Ng WF (2018). CD40 as a therapeutic target in Sjögren’s syndrome. Expert Rev. Clin. Immunol..

[65] Berner B, Wolf G, Hummel KM, Müller GA, Reuss-Borst MA (2000). Increased expression of CD40 ligand (CD154) on CD4+ T cells as a marker of disease activity in rheumatoid arthritis. Ann. Rheum. Dis..

[66] Voigt EA (2019). Sex differences in older adults’ immune responses to seasonal influenza vaccination. Front. Immunol..

[67] Xie H (2011). 17β-estradiol induces CD40 expression in dendritic cells via MAPK signaling pathways in a mini chromosome maintenance protein 6-dependent manner. Arthritis Rheum..

[68] Vaeth M (2014). Follicular regulatory T cells control humoral autoimmunity via NFAT2-regulated CXCR5 expression. J. Exp. Med..

[69] Kawabe T, Matsushima M, Hashimoto N, Imaizumi K, Hasegawa Y (2011). CD40/CD40 ligand interactions in immune responses and pulmonary immunity. Nagoya J. Med. Sci..

[70] Durie FH (1993). Prevention of collagen-induced arthritis with an antibody to gp39, the ligand for CD40. Science.

[71] Gong Y, Tong J, Wang S (2017). Are follicular regulatory T cells involved in autoimmune diseases?. Front. Immunol..

[72] Indrevær RL (2015). IRF4 is a critical gene in retinoic acid-mediated plasma cell formation and is deregulated in common variable immunodeficiency-derived B cells. J. Immunol..

[73] Carreras E (2010). Estrogen receptor signaling promotes dendritic cell differentiation by increasing expression of the transcription factor IRF4. Blood.

[74] Myles A, Gearhart PJ, Cancro MP (2017). Signals that drive T-bet expression in B cells. Cell Immunol..

[75] Pellegrini P, Contasta I, Del Beato T, Ciccone F, Berghella AM (2011). Gender-specific cytokine pathways, targets, and biomarkers for the switch from health to adenoma and colorectal cancer. Clin. Dev. Immunol..

[76] Yan, H. *et al*. B lymphocytes are a major source of IL-27 that drives class-switched antibody responses and anti-viral immunity through paracrinic targeting of B cells and T follicular helper cells. *J. Immunol*. **200**(1 Supplement) 107.5 (2018).

[77] Miles B, Connick E (2019). Control of the germinal center by follicular regulatory T cells during infection. Front. Immunol..

[78] Du F (2008). T-614, a novel immunomodulator, attenuates joint inflammation and articular damage in collagen-induced arthritis. Arthritis Res. Ther..

[79] Ramos-Romero S (2012). Effect of a cocoa flavonoid-enriched diet on experimental autoimmune arthritis. Br. J. Nutr..

[80] Fossati-Jimack L (2000). Markedly different pathogenicity of four immunoglobulin G isotype-switch variants of an antierythrocyte autoantibody is based on their capacity to interact *in vivo* with the low-affinity Fcgamma receptor III. J. Exp. Med..

[81] Mukherjee P (2003). TNF receptor gene therapy results in suppression of IgG2a anticollagen antibody in collagen induced arthritis. Ann. Rheum. Dis..

[82] Nandakumar KS, Holmdahl R (2007). Collagen antibody induced arthritis. Methods Mol. Med..

