# Supplementary material for: Sex differences in Tfh cell help to B cells contribute to sexual dimorphism in severity of rat collagen-induced arthritis
Source: Sci Rep. 2020 Jan 27;10:1214. doi: 10.1038/s41598-020-58127-y (PMC6985112; doi:10.1038/s41598-020-58127-y)
Supplement: Supplementary file 1 — Dataset 1. [file 41598_2020_58127_MOESM1_ESM.pdf]

Sex differences in Tfh cell help to B cells contribute to sexual dimorphism in severity of rat collagen-induced arthritis

Mirjana Dimitrijević[a], Nevena Arsenović-Ranin[b], Duško Kosec[c], Biljana Bufan[b], Mirjana Nacka-Aleksić[d], Ivan Pilipović[c], Gordana Leposavić[d*]

[a]Department of Immunology, Institute for Biological Research "Siniša Stanković", University of Belgrade, Bulevar despota Stefana 142, Belgrade, Serbia

[b]Department of Microbiology and Immunology, Faculty of Pharmacy, University of Belgrade, Vojvode Stepe 450, Belgrade, Serbia

[c]Immunology Research Center "Branislav Janković", Institute of Virology, Vaccines and Sera "Torlak", Vojvode Stepe 458, Belgrade, Serbia

[d]Department of Pathobiology, Faculty of Pharmacy, University of Belgrade, Vojvode Stepe 450, Belgrade, Serbia

Supplementary Table S1. Antibodies and reagents/second step reagents used in this study for flow cytometry analysis

**Antibodies and reagents**	**Company**
PE-conjugated anti-CD4 (clone OX-38)	BD Biosciences (USA)
APC-conjugated anti-CD4 (clone OX35)	eBioscience (USA)
PE/Cy5-conjugated anti-CD45RA (clone OX-33)	BD Biosciences
PerCP-conjugated anti-TCRαβ (clone R73)	BD Biosciences
Alexa Fluor 647-conjugated anti-TCRαβ (clone R73)	Biolegends (USA)
Anti-CXCR5 (clone EPR8837)	Abcam (UK)
FITC-conjugated anti-CD40 (clone HM40-3)	BD Biosciences
Anti-CD40L (CD154) (clone B-4)	Santa Cruz Biotechnology (USA)
PerCP-eFluor® 710-conjugated anti-CD25 (clone OX39)	eBioscience
Biotin-conjugated anti-IgM (clone MRM-47)	Biolegends
PE-conjugated anti-IL-17A (clone TC11-18H10)	BD Biosciences
FITC-conjugated anti-IFN-γ (clone DB-1)	BD Biosciences
PE-conjugated anti-IL-4 (clone OX-81)	BD Biosciences
FITC-conjugated anti-Ki-67 (clone B56)	BD Biosciences
FITC-conjugated anti-Foxp3 (clone FJK-16s)	eBioscience
Anti-IRF4 polyclonal antibody	ThermoFisher Scientific (USA)
FITC-conjugated Annexin V	BD Biosciences
FITC-conjugated streptavidin	BD Biosciences
FITC-conjugated goat anti-rabbit IgG	BD Biosciences
FITC-conjugated goat anti-mouse IgG	BD Biosciences
Donkey anti-rabbit F(ab')2 IgG	Abcam

FITC-fluorescein isothiocyanate; PE-phycoerythrin; APC-allophycocyanin; PerCP-peridinin–chlorophyll–protein; Cy-Cyano dye.

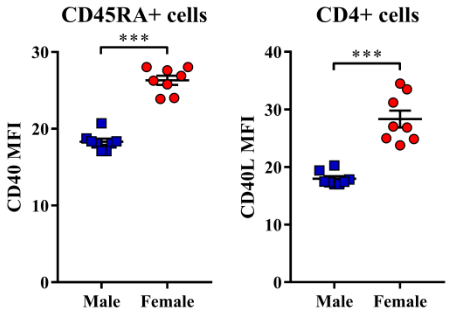

Supplementary Figure S1. Sex differences in the expression levels for CD40 on CD45RA+ cells and CD40L on CD4+ cells in male and female CIA rats. Cells were obtained from lymph nodes draining inflamed joints and nearby tissues (dLNs) recovered at the peak of the clinical severity of CIA. Scatter plots show the mean fluorescence intensity (MFI) for the expression of CD40 on CD45RA+ cells (left) and CD40L on CD4+ cells in dLN cells from male and female rats. Horizontal lines within scatter plots indicate mean values. n = 8 rats per group. *** $p < 0.001$.

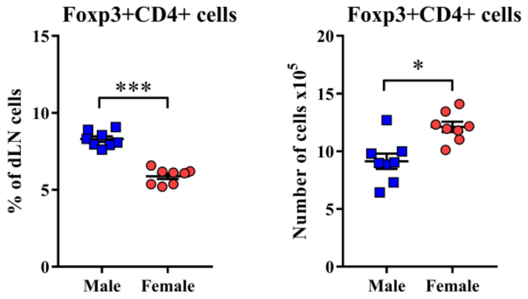

Supplementary Figure S2. Sex differences in the generation of Foxp3+CD4+ cells in male and female CIA rats. Cells were obtained from lymph nodes draining inflamed joints and nearby tissues (dLNs) recovered at the peak of the clinical severity of CIA. Scatter plots show the frequency (left) and the number (right) of Foxp3+CD4+ cells among dLN cells from male and female rats. Horizontal lines within scatter plots indicate mean values. n = 8 rats per group. * $p < 0.05$, *** $p < 0.001$.

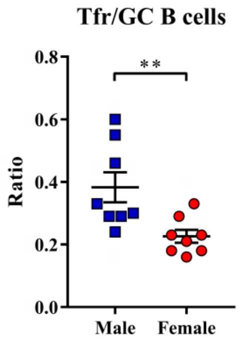

Supplementary Figure S3. Sex difference in Tfr/GC B cells ratio in rat CIA. Cells were obtained from lymph nodes draining inflamed joints and nearby tissues (dLNs) recovered at the peak of the clinical severity of CIA. Scatter plot shows Tfr/Germinal Center (GC) B cell ratio in dLNs of male and female rats. Data are presented as means ± SEM. Horizontal lines within scatter plots indicate mean values. n = 8 rats per group. **$p < 0.01$.

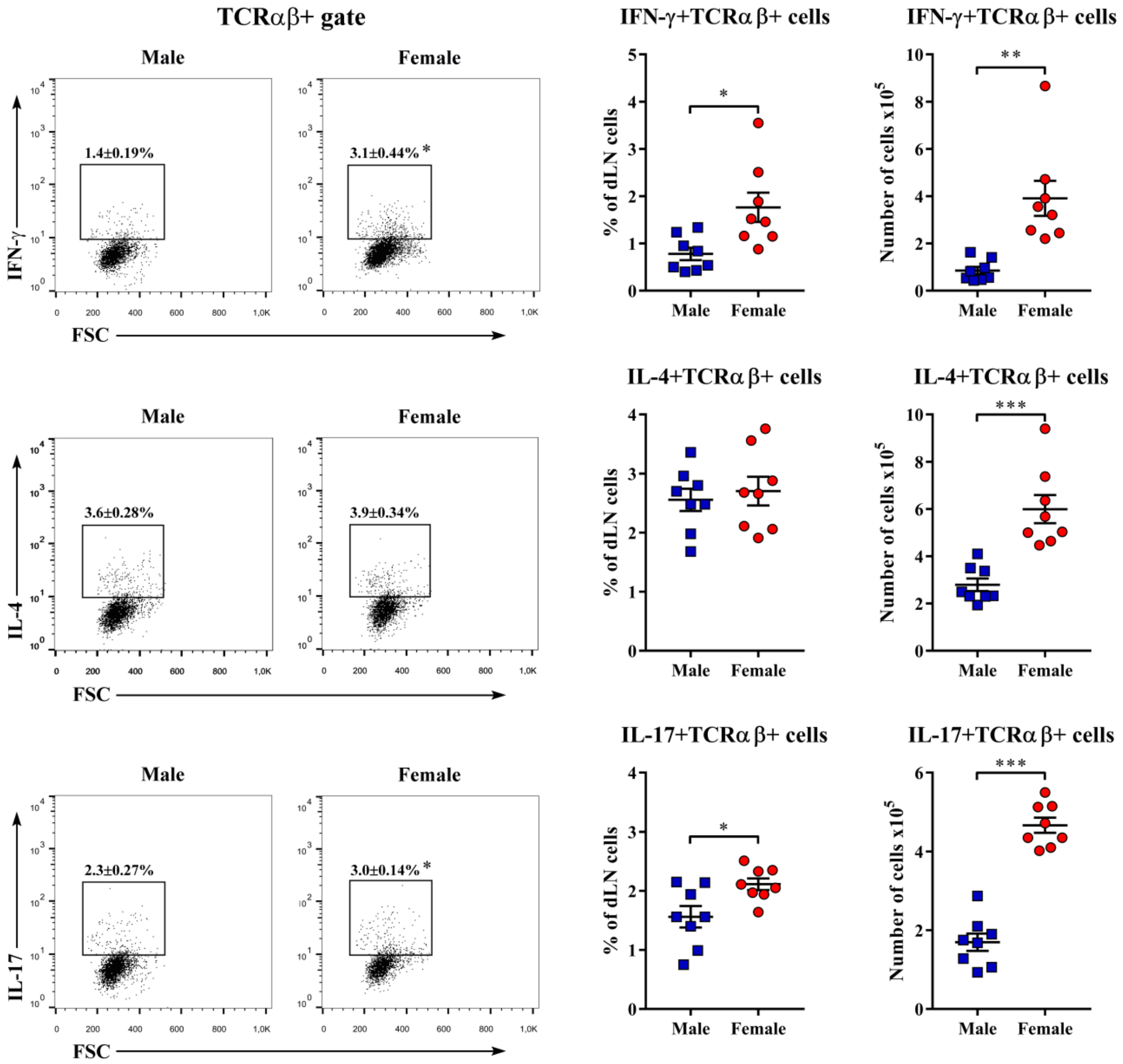

Supplementary Figure S4. Sex differences in the generation of T cells which produce cytokines shaping CII-specific IgG subclass antibody profile in rat CIA. Cells were obtained from lymph nodes draining inflamed joints and nearby tissues (dLNs) recovered at the peak of the clinical severity of CIA. Representative flow cytometry dot plots show IFN-γ, IL-4 and IL-17 staining of TCRαβ+ cells from dLNs of male and female rats. The number indicates the percentage (mean ± SEM) in the region. Scatter plots show the percentage of IFN-γ+TCRαβ+, IL-4+TCRαβ+ and IL-17+TCRαβ+ cells from dLNs of male and female rats. Data are presented as means ± SEM. Horizontal lines within scatter plots indicate mean values. n = 8 rats per group. *p < 0.05, **p < 0.01, ***p < 0.001.

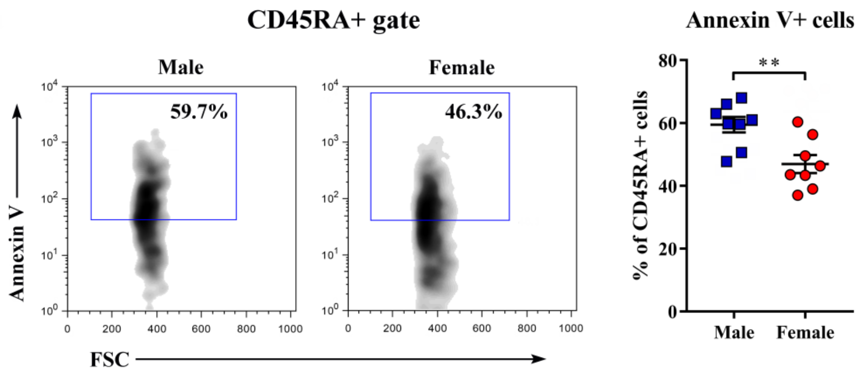

Supplementary Figure S5. Sex difference in apoptosis of B cells in rat CIA. Cells were obtained from lymph nodes draining inflamed joints and nearby tissues (dLNs) recovered at the peak of the clinical severity of CIA. Representative flow cytometry density plots show Annexin V staining of CD45RA+ cells from dLNs of male and female rats. The number indicates the percentage in the region. Scatter plots show the frequency of Annexin+ cells among CD45RA+ cells. Data are presented as means ± SEM. Horizontal lines within scatter plots indicate mean values. n = 8 rats per group. **$p < 0.01$.
